# Morphological Evaluation and Immunohistochemical Analysis of the Reparative Potential of the Buccal Fat Pad

**DOI:** 10.3390/medicina60040567

**Published:** 2024-03-30

**Authors:** Roman Zhidkov, Andrew Panin, Aleksei Drobyshev, Tatiana Demura, Sofya Avraamova, Petr Aleksandrov, Anastasia Kolesnikova, Hadi Darawsheh, Anna Turkina, Nicolai Redko, Yaroslav Skakunov, Elena Karpova, Anzhela Brago, Aleksandr Tsitsiashvili, Yuriy Vasil’ev

**Affiliations:** 1 Federal State Budgetary Educational Institution of Higher Education «ROSUNIMED» of the Ministry of Health of the Russian Federation, 119991 Moscow, Russia; orthosurg@yandex.ru (R.Z.); profpanin@gmail.com (A.P.); dr.drobyshev@gmail.com (A.D.); amc777@yandex.ru (A.T.); 2 Institute of Clinical Morphology and Digital Pathology, I.M. Sechenov First Moscow State Medical University, 119435 Moscow, Russia; demura_t_a@staff.sechenov.ru (T.D.); avraamova_s_t@staff.sechenov.ru (S.A.); aleksandrov_p_s@staff.sechenov.ru (P.A.); kolesnikova_a_o@student.sechenov.ru (A.K.); 3 N.V. Sklifosovskiy Institute of Clinical Medicine, I.M. Sechenov First Moscow State Medical University, 119435 Moscow, Russia; daraushe_kh_m@staff.sechenov.ru; 4 E.V. Borovsky Institute of Dentistry, I.M. Sechenov First Moscow State Medical University, 119435 Moscow, Russia; turkina_a_yu@staff.sechenov.ru; 5 Federal State Budgetary Educational Institution of Higher Education, Pirogov Russian National Research Medical University of the Ministry of Health of the Russian Federation, 119991 Moscow, Russia; elena-karpova@inbox.ru; 6 Department of Propedeutics of Dental Diseases, Medical Institute, Peoples’ Friendship University of Russia Named after Patrice Lumumba, 117198 Moscow, Russia; anzhela_bogdan@mail.ru

**Keywords:** oroantral communication, buccal fat pad, mesenchymal stem cells, immunohistochemistry, morphology

## Abstract

*Background and Objectives*: There are many surgical techniques for oroantral communication treatment, one of which is the buccal fat pad. Of particular interest is the high reparative potential of the buccal fat pad, which may be contributed to by the presence of mesenchymal stem cells. The purpose of this work is to evaluate the reparative potential of BFP cells using morphological and immunohistochemical examination. *Materials and Methods*: 30 BFP samples were provided by the Clinic of Maxillofacial and Plastic Surgery of the Russian University of Medicine (Moscow, Russia) from 28 patients. Morphological examination of 30 BFP samples was performed at the Institute of Clinical Morphology and Digital Pathology of Sechenov University. Hematoxylin–eosin, Masson trichrome staining and immunohistochemical examination were performed to detect MSCs using primary antibodies CD133, CD44 and CD10. *Results*: During staining with hematoxylin–eosin and Masson’s trichrome, we detected adipocytes of white adipose tissue united into lobules separated by connective tissue layers, a large number of vessels of different calibers, as well as the general capsule of BFP. The thin connective tissue layers contained neurovascular bundles. Statistical processing of the results of the IHC examination of the samples using the Mann–Whitney criterion revealed that the total number of samples in which the expression of CD44, CD10 and CD133 antigens was confirmed was statistically significantly higher than the number of samples where the expression was not detected (*p* < 0.05). *Conclusions*: During the morphological study of the BFP samples, we revealed statistically significant signs of MSCs presence (*p* < 0.05), including in the brown fat tissue, which proves the high reparative potential of this type of tissue and can make the BFP a choice option among other autogenous donor materials when eliminating OAC and other surgical interventions in the maxillofacial region.

## 1. Introduction

There are many surgical techniques for oroantral communication (OAC) treatment that utilize different groups of materials [1,2], but the gold standard is considered to be the use of autogenous tissue, one of which is the buccal fat pad (BFP).

The buccal fat pad is represented by a paired encapsulated fat formation. On the one hand, it has biological implementation at different ages of the patient, and on the other hand, it can be used in small and large reconstructive surgical procedures, especially in the maxillofacial region [3]. These include the repair of oral cavity defects after resective surgeries, closure of clefts of the hard and soft palate, treatment of peri-implantitis and repair of defects around dental implants, and the management of tooth recessions and OAC [4], as well as in the treatment of temporomandibular joint ankylosis [5,6,7,8,9], congenital pathologies, jawbone necrosis, and postoperative reconstructions in cancer patients [10]. 

In addition to anatomical proximity to the surgical area and good plastic properties, the BFP is abundantly supplied with blood, which allows us to speak both about undesirable events in case of injuries and about the high reparative potential of this tissue type. Of particular interest is the possible presence of mesenchymal stem cells (MSCs) in the BFP, especially in its most vascularized part—brown fat [11,12].

In addition to accelerating soft tissue regeneration of the wound area, this cell type can be converted into bone tissue, which may allow not just the repair of the postoperative defect, but the restoration of a whole complex of lost tissues in the region of OAC, as evidenced by tissue engineering data [13]. 

According to the authors of [14], the BFP and its cellular composition contributed to optimal bone density formation for further surgery: there was a more equal marginal fusion with enhanced bone trabecular formation and well-organized and vascularized lamellar bone with haversian canals and osteocytes. The authors conclude that the high quality of the newly formed bone met the specified criteria for function and quality, improving quality of life and reducing secondary complications. The publications of [7,15] discuss the use of a flap containing BFPs in such surgeries as scar elimination, treatment of hemifacial microsomia and temporal skeletonization. From a retrospective point of view, a systematic review of data from 2004 to 2009 in bibliographic databases is interesting [16]: the authors indicate the anatomical advantages of BFPs and the ergonomics of their intraoperative use, both in working with bone and with soft tissue defects. Of particular interest is the volumization of the facial region after orthognathic surgeries for subcutaneous fat deficiency [17]. The authors of [18] showed that BFP application can be used to prevent fistula formation after palatal plasty, wound contracture and, subsequently, velopharyngeal insufficiency with potential hypoplasia of the middle part of the face. At the same time, mobilization of the BFP from intraoral access does not significantly change the facial configuration [19].

When performing orthognathic surgeries on the maxilla, especially if the patient wishes to perform a midface plasty, a part of the BFP can be removed. 

In the morphological structure of the BFP, connective and adipose tissue are distinguished, and a few different authors describe not only white fat, but also brown fat [11,20].

According to Sbarbati et al. (2010), there are three types of white adipose tissue (WAT) that can be differentiated based on structural and ultrastructural features: deposit WAT (dWAT), structural WAT (sWAT) and fibrous WAT (fWAT) [21].

Thus, according to Sbarbati et al. (2010), deposit white adipose tissue cells are tightly packed and linked by a weak net of isolated collagen fibers. The collagenic components are very poor, the cells are large and few blood vessels are present. The deep portion appears more fibrous than the superficial one. Microcirculation is formed by thin-walled capillaries with rare stem niches. Reinforcement pericyte elements are rarely evident. The same study describes the features of structural white adipose tissue: the stroma is well represented, with good vascularity and adequate staminality; cells are wrapped by a basket of collagen fibers; and the fatty depots of the knees and the trochanteric areas have quite loose meshes. Finally, fibrous white adipose tissue has a noteworthy fibrous component.

According to Saely et al. (2012), white and brown adipose tissue have different origins—white fat is from Myf5-negative progenitor cells, and brown fat is from Myf5-positive progenitor cells—which once again emphasizes the significant difference between these tissues and indicates a functional antagonism. In their study, the authors describe the histological structure of both tissues: white is represented by a single lipid droplet and multiple small vacuoles, and brown is represented by a variable number of mitochondria and abundant mitochondria [22].

Evidencing the presence of MSCs in the BFP may increase its value as an autologous donor material and make it an option of choice when planning OAC management, which determined the purpose of this work.

The purpose of this work is to evaluate the reparative potential of BFP cells using morphological and immunohistochemical examinations. 

## 2. Materials and Methods

A total of 30 BFP samples were provided by the Clinic of Maxillofacial and Plastic Surgery of the Russian University of Medicine (Moscow, Russia) from 28 patients after obtaining informed voluntary consent: 10 men and 18 women aged 19 to 42 years (median 26 years). The BFP volume in one patient was so extensive that it allowed for multiple fragments to be taken, increasing the total to 30. Patients were informed about the technique of surgical intervention using BFPs; possible complications; and alternative treatment options, their advantages, and disadvantages (ethics committee report—01-22 from 20 January 2022). A fragment of the buccal fat pad was harvested intraoperatively during orthognathic maxilla surgery with Le-Fort I osteotomy [23]. If an excessive amount of BFP was found in the operative area and prevented proper alignment of the maxilla’s parts, part of it was removed using surgical scissors.

We determined the sample size using the formula

SS = Z^2^ × (p) × (1 − p)/C^2^,

where Z = Z factor (1.96 at 95% confidence interval), p = percentage of respondents of interest or responses in decimal form (0.5 by default), and c = confidence interval in decimal form (0.05 = ±5%).

The sample size calculation for a general population of 30 showed a required sample size of 28. Given the data obtained, the margin of error or confidence interval was ±4.86%, which did not change the final sample size calculation. 

The obtained BFP samples were placed in a container with 10% buffered neutral formalin solution (HISTOSAFE, Russian Federation) at room temperature and then put in the fridge at +4 °C.

Morphological examination of the 30 BFP samples was performed at the Institute of Clinical Morphology and Digital Pathology of Sechenov University. Hematoxylin–eosin staining according to the classical technique and Masson’s trichrome [24] staining was performed for the general evaluation of tissue and cellular structures, and an immunohistochemical study (IHC) of the samples was performed to evaluate the reparative potential of the BFP, namely the detection of MSCs using primary antibodies. 

Fixation and histological processing of the material were performed according to the standard protocol. Then, the material was embedded in paraffin and serial paraffin sections 3–4 µm thick were made. For histological study, the sections were stained with hematoxylin and eosin, and Masson’s trichrome. 

### Immunohistochemical Method

For IHC examination, serial paraffin sections 3–4 µm thick were placed on adhesive-coated glasses (Menzel Glaser Polylisine, Braunschweig, Germany). Unstained sections were processed using a standard immunohistochemical diagnostic method with thermal demasking of antigens. Demasking was performed in a specialized water bath PT Module (Thermo Scientific, Waltham, MA, USA): polylysine glass with paraffin sections were deparaffinized according to the standard method, and after rinsing in distilled water were immersed in containers with universal buffer for additional deparaffinization, demasking and rehydration of Trilogy slices (Cell Marque, Rocklin, CA, USA) and heated in a water bath to 95 °C for 20 min. The slides were then cooled at room temperature for 20 min. All further steps of immunohistochemical reaction were performed in a SlideMaster wet chamber (Bio Optica, Milan, Italy) to prevent drying of the sections. To block endogenous peroxidase, the slides were incubated for 10 min with a peroxidase inhibitor, after which the slices were rinsed in phosphate buffer (pH 7.0–7.6) (Cell Marque, Rocklin, CA, USA) and incubated with Ultra V Block (LabVision, Fremont, CA, USA) for 30 min to block nonspecific protein interactions. At the end of incubation, the excess reagent was gently shaken off the slides and primary antibodies were applied.

Mouse monoclonal antibodies CD44 (clone SD391, Xiamen Talent Biomedical, Xiamen, China, RTU), CD10 (clone GM003, PrimeBioMed, Moscow, Russia, dilution 1:100) and rabbit polyclonal antibodies to CD133 (Huabio, Woburn, MA, USA, dilution 1:500) were used as primary antibodies (Table 1).

To visualize the antibody–antigen binding site, the reaction of substrate oxidation by 3,3-diaminobenzidine (DAB) with horseradish peroxidase in the presence of hydrogen peroxide was used with the formation of an insoluble inorganic solvent reaction product, which was visible as brown staining of specific cell structures (N-Histofine^®^ DAB-2V, Nichirei, Tokyo, Japan).

Slices were incubated with DAB for 5 min to achieve the desired staining intensity. Then, the slides were washed in distilled water and the nuclei were stained with Mayer’s hematoxylin for 2–3 min. The slides were then dehydrated in a battery of ascending alcohols (70%, 80%, 95%, and absolute alcohol) and 3 xylenes. Slices were then coverslipped using BioMount synthetic medium (Bio Optica, Italy).

The CD44 antigen is expressed as brown staining of the cell membrane of hematopoietic cells with signs of stemness [25]. The CD133 antigen is expressed as brown staining of the membrane of cells with stemness features [26]. The CD10 antigen is expressed as brown staining of the cell membrane of brown adipocyte cells [27]. 

Positive and negative controls were obligatory for immunohistochemical reactions. As negative controls, samples of the studied sections were taken, which were subjected to the standard procedure of IHC investigation, but without incubation with primary antibodies. Positive controls for each antibody were selected according to the recommendations of the antibody manufacturers.

Histological samples were studied by light microscopy using a “Leica DM2500” microscope (Leica Microsystems, Wetzlar, Germany), with a magnification of ×100, ×200, ×400, and ×1000.

The degree of antigen expression in the IHC analysis was determined by the ratio of stained cells to the total amount of cells in 5 fields of view of 0.05 mm^2^ (total area—0.25 mm). We considered 15%—extremely intense expression, 10%—intense expression, 5%—moderate expression, 1%—weak expression, and 0%—no expression.

The data of the study results were entered into Microsoft Excel 16.3 (MacOS) spreadsheets. Statistical processing was performed using the Mann–Whitney criterion of the Stat Soft Statistica 10.0 program package for Windows.

## 3. Results

### 3.1. Hematoxylin–Eosin

**Figure 1:**
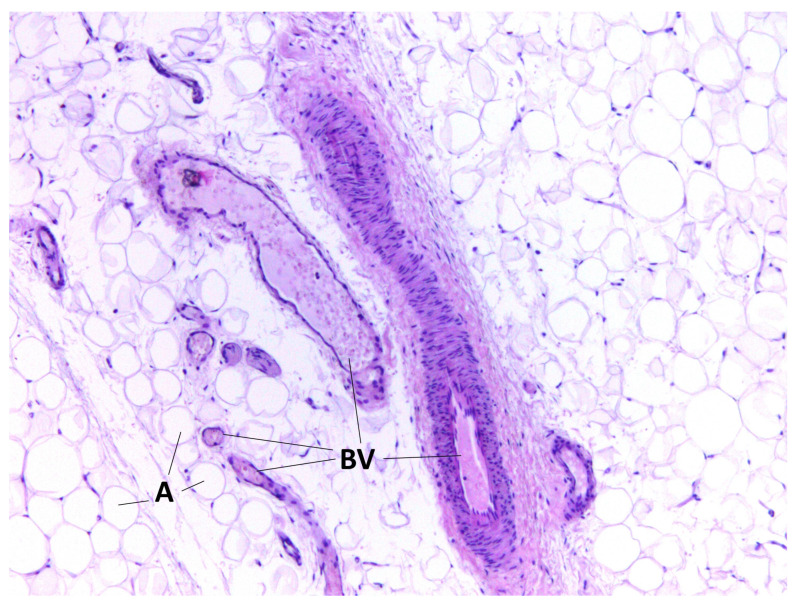
BFP sample, hematoxylin–eosin staining, magnification ×200. Adipocytes of white adipose tissue (A) and vessels of different calibers (BV) are visualized.

**Figure 2:**
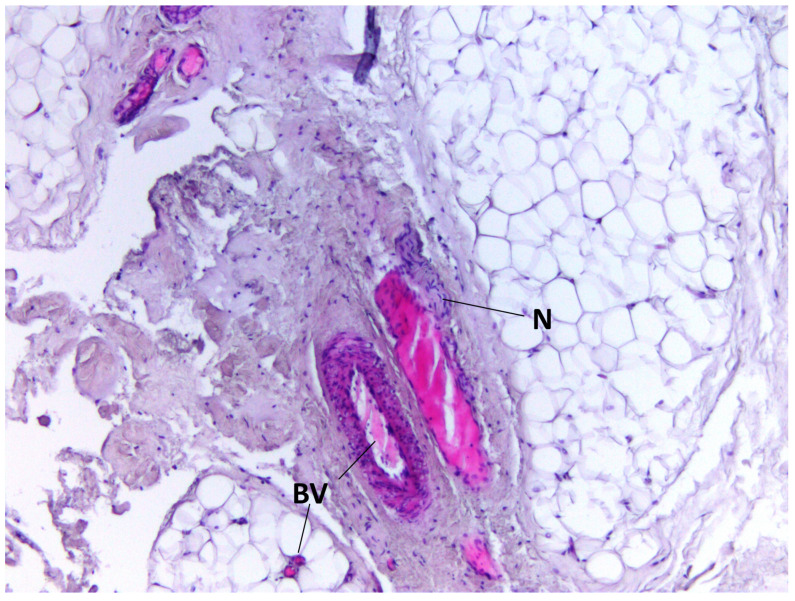
BFP sample, hematoxylin–eosin staining, magnification ×200. Blood vessels (BV) and nerve fiber (N) are visualized.

**Figure 3:**
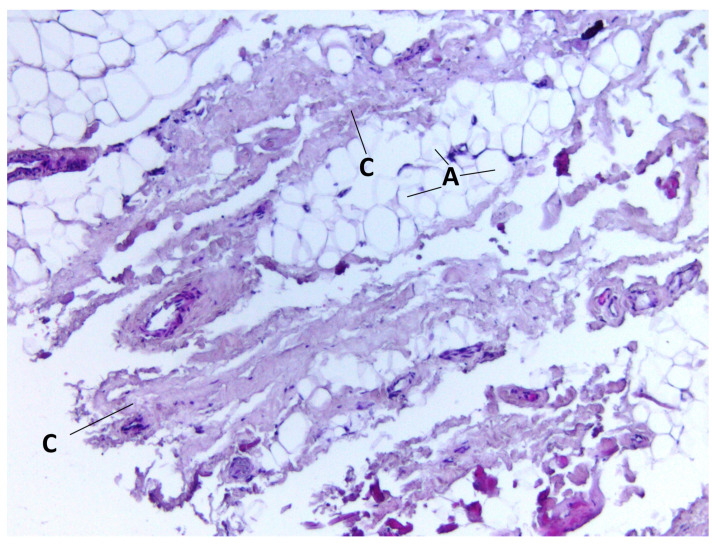
BFP sample, hematoxylin–eosin staining, magnification ×200. Adipocytes of adipose tissue (A) and BFP capsule (C) are visualized.

**Figure 4:**
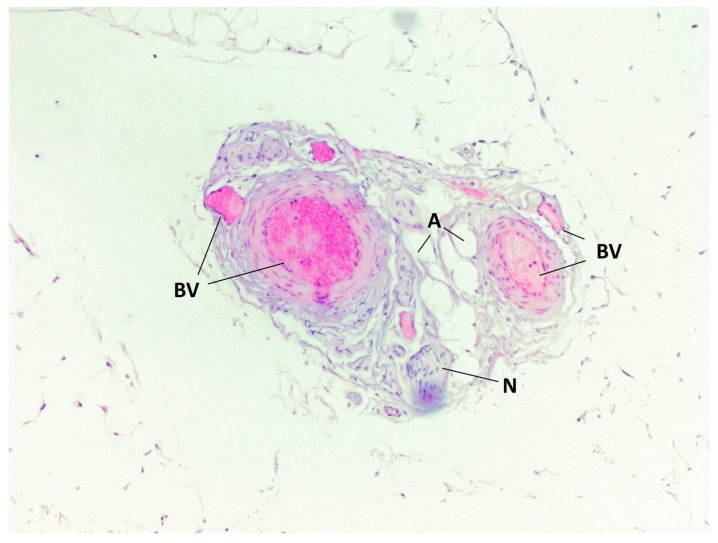
BFP sample, hematoxylin–eosin staining, magnification ×200. Adipocytes of adipose tissue (A), blood vessels (BV) and nerve fiber (N) are visualized.

During staining with hematoxylin–eosin dye according to the classical technique, we detected adipocytes of white adipose tissue united into lobules separated by connective tissue layers. Furthermore, in the BFP samples, we visualized a large number of vessels of different calibers, as well as the general capsule of BFP. The thin connective tissue layers contained neurovascular bundles. Cell clusters were found around the vessels (Figure 1, Figure 2, Figure 3 and Figure 4).

### 3.2. Masson’s Trichrome

**Figure 5:**
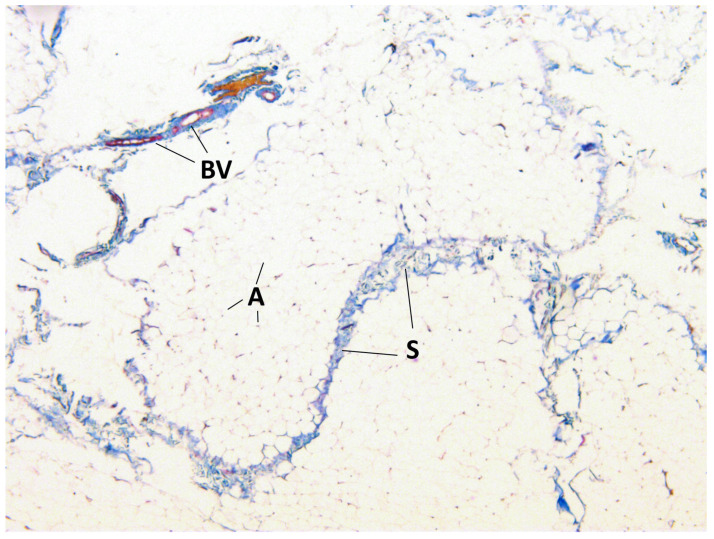
BFP sample, Masson’s trichrome staining, magnification ×100. Adipocytes (A), blood vessels (BV) and connective tissue septa between lobules (S) are visualized.

**Figure 6:**
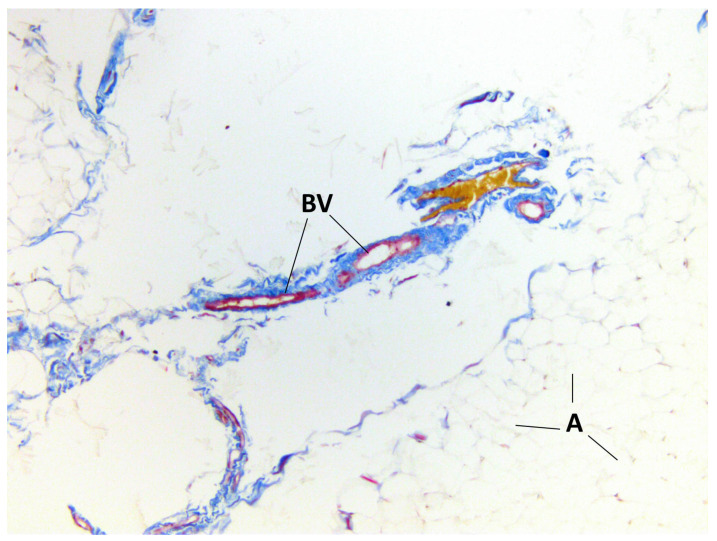
BFP sample, Masson’s trichrome staining, magnification ×200. Adipocytes (A) and blood vessels (BV) are visualized.

**Figure 7:**
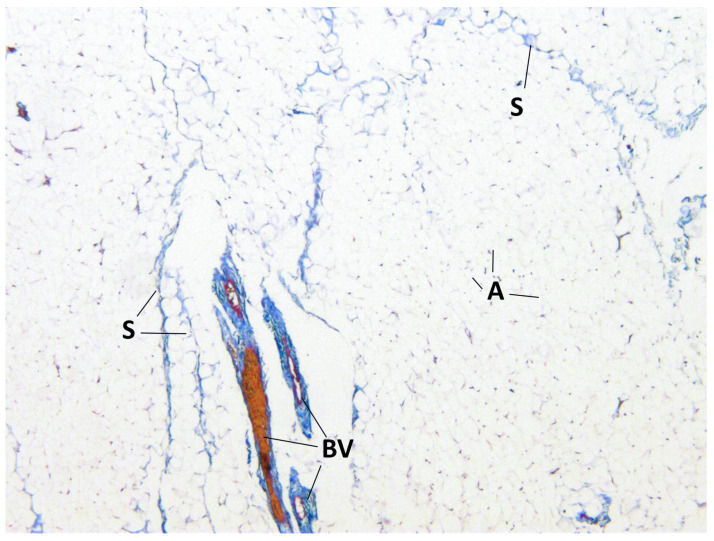
BFP sample, Masson’s trichrome staining, magnification ×100. Adipocytes (A), blood vessels (BV) and connective tissue septa between the lobules (S) are visualized.

The presence of connective tissue layers between the lobules of BFP and the common capsule was confirmed by staining the samples (Figure 5, Figure 6 and Figure 7).

### 3.3. IHC Study

#### 3.3.1. CD44

**Figure 8:**
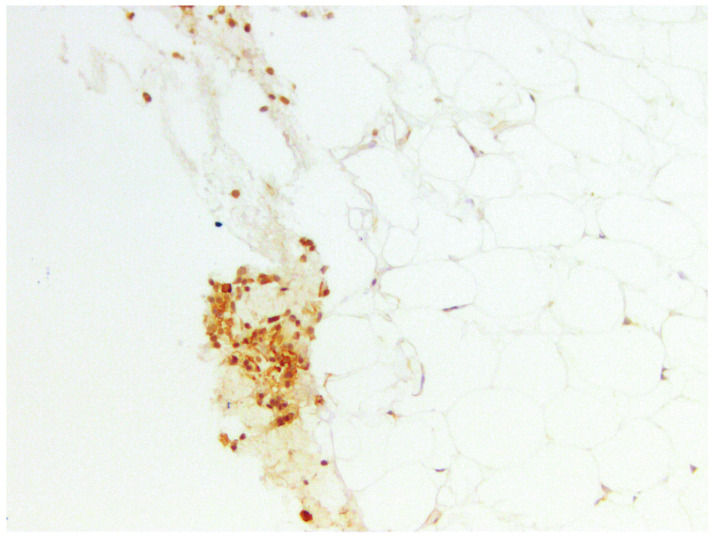
CD44 in BFP sample, IHC reaction, hematoxylin counterstaining. ×200.

**Figure 9:**
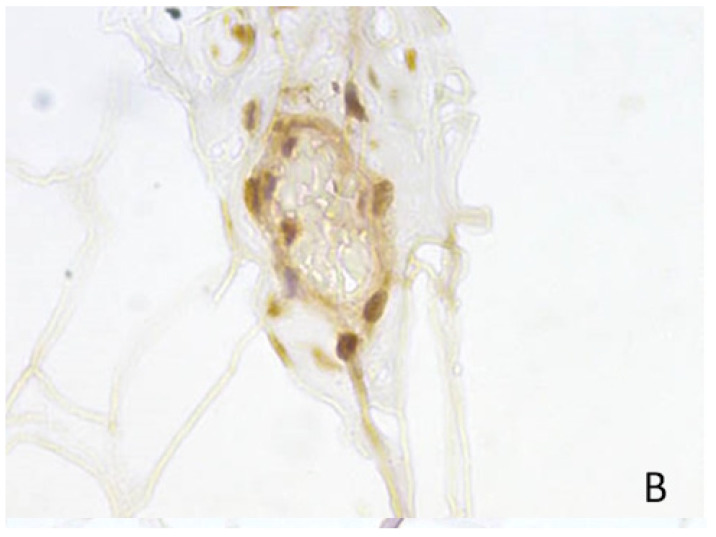
CD44 in BFP sample, IHC reaction, hematoxylin counterstaining. ×1000.

We identified CD44-positive cells in brown fat areas in the buccal fat pad of the studied patients, with positive cells localized predominantly in the perivascular space (Figure 8 and Figure 9). We propose that MSC localization is limited to growth zones in the tissues that are localized around capillaries and in the brown fat tissue.

**Figure 10:**
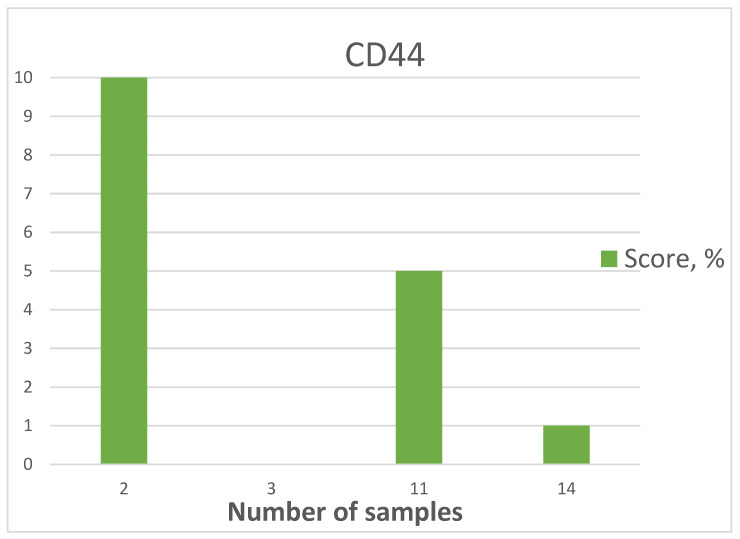
CD44 expression diagram.

The highest expression was detected in two samples and amounted to 6.67% (Figure 8), moderate expression was detected in eleven samples (36.67%) (Figure 9), low expression was detected in fourteen samples (46.67%), and no expression was detected in three samples (10%) (Figure 10).

**Figure 11:**
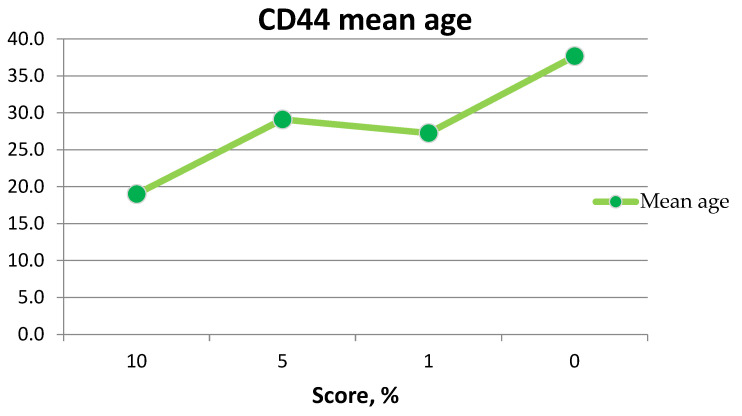
Graph of patients’ age with CD44 expression.

The mean age of patients showing CD44 expression was 27.4 years. The maximum age was 38 years, and the minimum age was 19 years (Figure 11, Table 2).

#### 3.3.2. CD133

**Figure 12:**
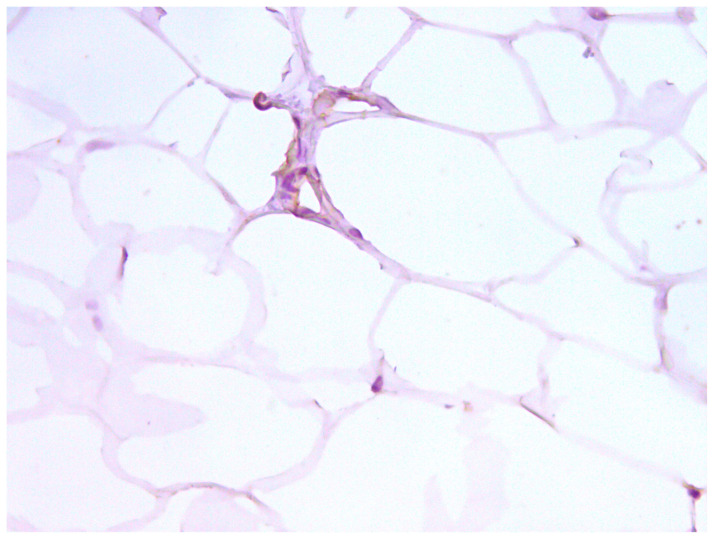
CD133 in BFP sample, IHC reaction, hematoxylin counterstaining. ×400.

**Figure 13:**
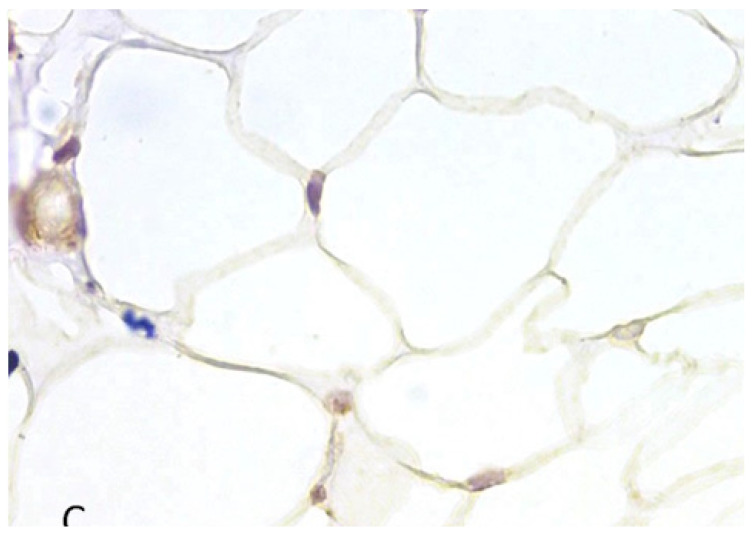
CD133 in BFP sample, of IHC reaction, hematoxylin counterstaining. ×1000.

CD133-positive cells were found in small areas of the buccal fat pad of the studied patients, which corresponded in localization to sites with a presence of growth zones of brown fat (Figure 12 and Figure 13).

**Figure 14:**
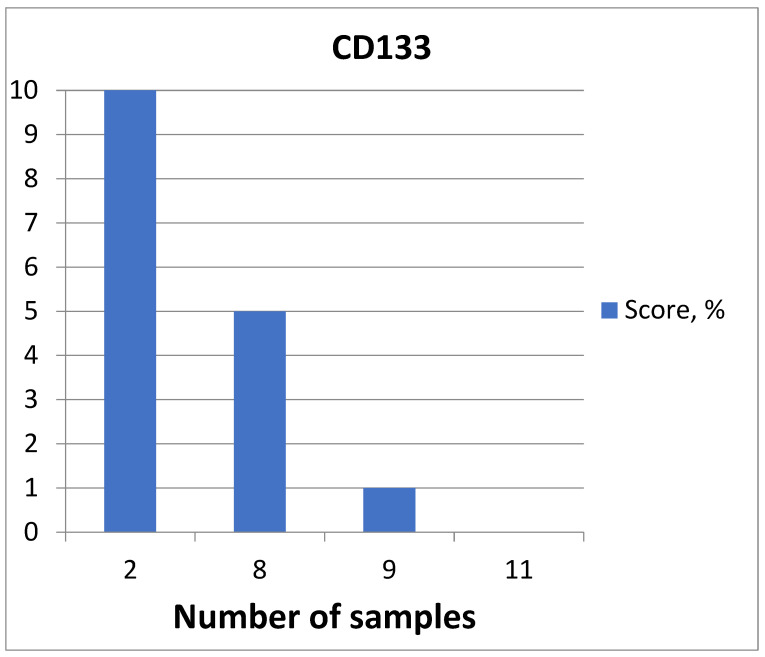
CD133 expression diagram.

The highest expression was detected in two patients (6.67%) (Figure 13), moderate expression was detected in eight patients (26.67%) (Figure 12), low expression was detected in nine patients (30%), and no expression was detected in eleven patients (36.67%) (Figure 14).

**Figure 15:**
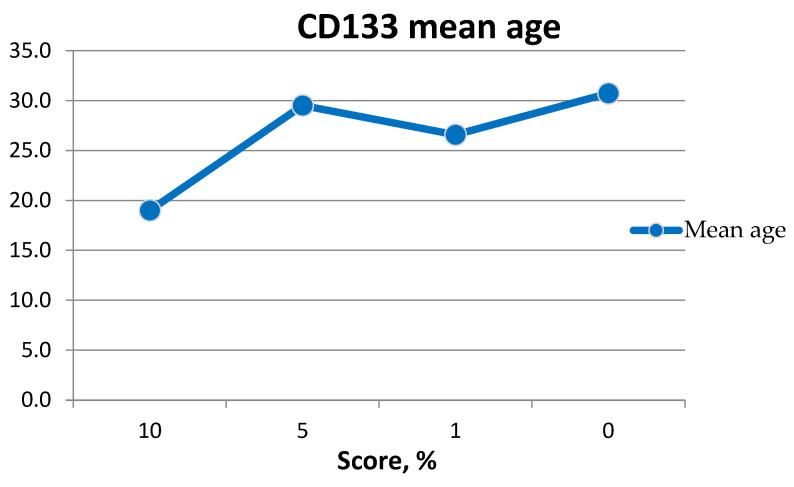
Graph of patients’ age with CD133 expression.

The mean age of patients showing CD133 expression was 27 years (Figure 15). The maximum age was 38 years, and the minimum age was 19 years (Table 3).

#### 3.3.3. CD10

**Figure 16:**
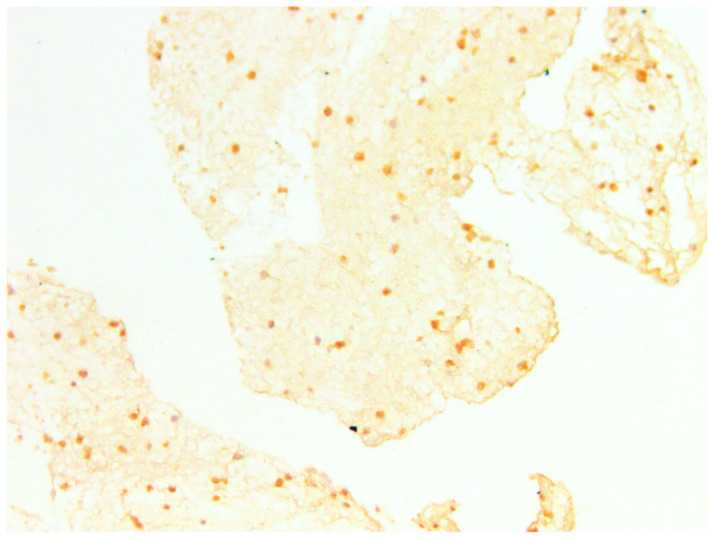
CD10 in BFP sample, IHC reaction, hematoxylin counterstaining. ×100.

**Figure 17:**
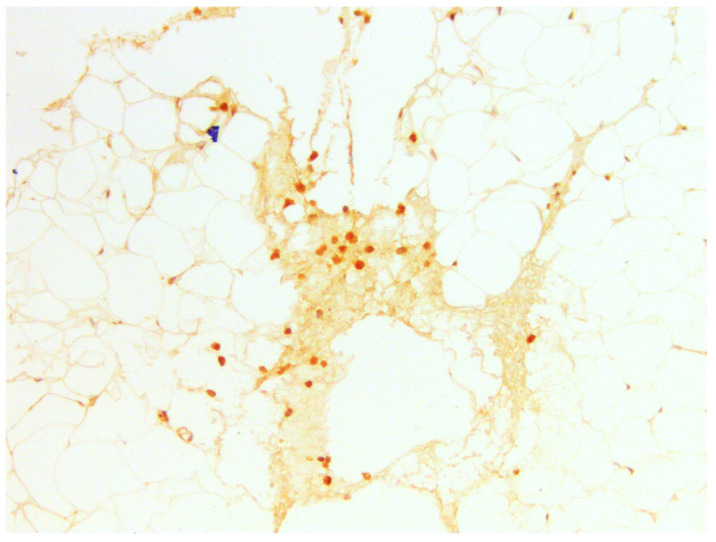
CD10 in small region of BFP sample, IHC reaction, hematoxylin counterstaining. ×200.

According to the authors (Gjorgova-Gjeorgjievski S, 2021, [27]), CD10 is expressed in brown fat, which is confirmed by previous markers. When comparing samples—photos taken from the same regions—there was colocalization of CD44 and CD133 in BFPs positive for CD10 (Figure 16 and Figure 17). Colocalization of CD44 and CD133 was found in similar locations—in zones of growth that are characterized by pericapillary regions and especially in brown fat tissue. We supported our findings by using a series of sections that were stained sequentially by hematoxylin and eosin, Masson’s trichrome, and immunohistochemical markers.

**Figure 18:**
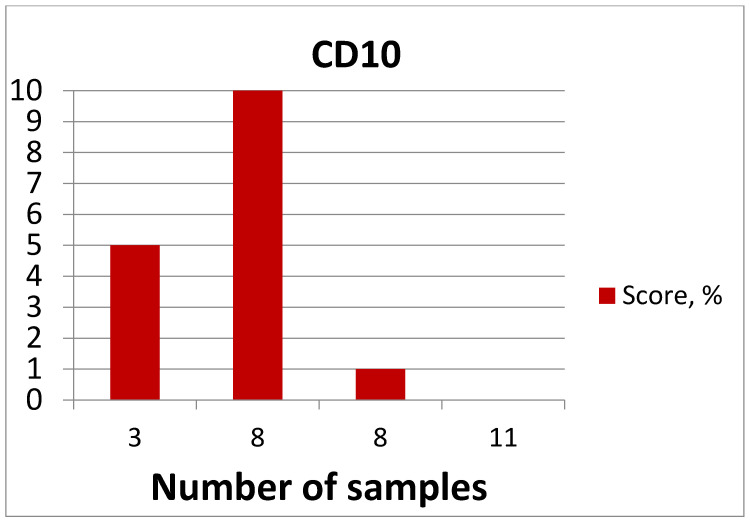
CD10 expression diagram.

The highest expression was detected in eight patients (26.67%), moderate expression was detected in three patients (10%), low expression was detected in eight patients (26.67%), and no expression was detected in eleven patients (36.67%) (Figure 18).

**Figure 19:**
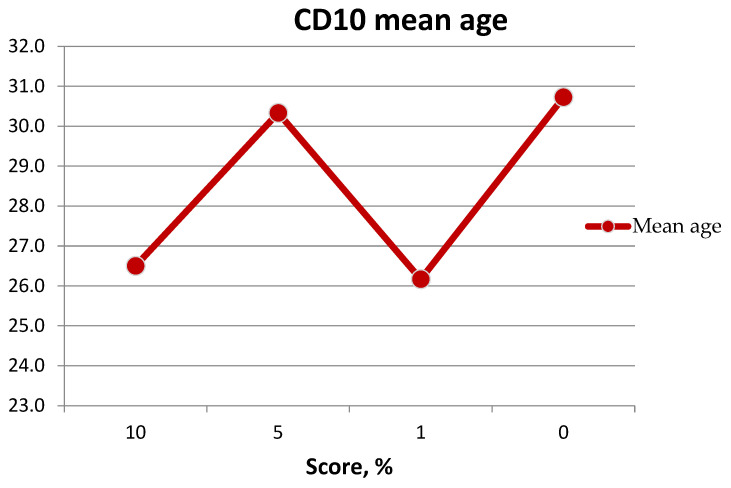
Graph of patients’ age with CD10 expression.

The mean age of patients showing CD10 expression was 27 years. The maximum age was 38 years, and the minimum age was 19 years (Figure 19, Table 4).

Patients with minimal antigen expression are mostly older than 30 years, but there are some exceptions (a 38-year-old patient and a 34-year-old patient). The youngest patient—19 years old—had maximum expression of all antigens.

Statistical processing of the results of the IHC examination of the samples using the Mann–Whitney criterion revealed that the total number of samples in which the expression of CD44, CD10 and CD133 antigens was confirmed (27–90%, 19–63.3% and 19–63.3%, respectively) was statistically significantly higher than the number of samples where the expression was not detected (*p* < 0.05).

## 4. Discussion

The investigated markers are detected in lymphohematopoietic precursor cells (CD44 and CD133), adipose tissue stem cells and brown fat cells (CD10) [26,27,28]. 

CD10, CD44 and CD133 were detected in small but significant amounts in brown fat in perivascular spaces, and within brown fat lobules in individual cells that are limited to tissue growth zones. When comparing the localization of the markers, their concomitant detection in the same areas mentioned above was noted.

In 19 samples (63.3%), there was CD133-positive staining, which also indicates the presence of somatic stem cells. There was also CD10 brown adipose tissue in 19 samples (63%). Most of all samples had CD44 expression—27 (90%)—which indicates the presence of stem cells in the composition of the BFP. These data are confirmed in the studies of other authors [29,30].

The BFP is a type of adipose tissue separate from the subcutaneous fat tissue. The main function of the BFP is to participate in infant sucking and to facilitate muscle gliding during mastication [7]. Being located predominantly in the midface area, the BFP is also involved in the formation of the aesthetic contour of the face [31]. In this regard, the main concern of practicing dental surgeons and maxillofacial surgeons may be a probable change in the facial configuration when using the BFP to repair defects in the oral cavity and maxillofacial region. However, according to the results of our experimental study and the authors’ data, extrusion of the BFP does not lead to changes in the face’s aesthetic parameters on the intervention side, especially when using its more deeply located extensions, such as the temporal one [19,32]. This procedure cannot be equated with a classical bichectomy.

The authors note the positive properties of the BFP as an autogenous donor material for surgical operations in the maxillofacial region, including the elimination of OAC—its anatomical proximity to the surgical area, high plasticity, as well as the ability to repair the lost tissue in the defect area and accelerated healing and epithelialization, even in open wound management—which is possible due to its high reparative potential [7,33,34,35,36].

There is information that brown adipose tissue of the BFP occupies a significant volume of the BFP only in the neonatal period, and then is almost completely replaced by white adipose tissue [20]; however, other authors claim that some areas of brown fat in the BFP can be detected in adults as well. In addition, areas with brown fat have the highest vascular density and are therefore an area potentially rich in mesenchymal stem cells [11]. The detection of antigen colocalization zones in our work confirms this hypothesis. 

Being essentially pluripotent MSCs, they are precursor cells for various types of connective tissue. It is reported that the MSCs of the BFP can be transformed into white adipose tissue and cartilage tissue, as well as into bone tissue, and in in vitro experiments the authors were able to stimulate the formation of tissue like human dental dentin from BFP MSCs [33,37,38]. Other in vitro studies have compared the bone formation capacity of different types of mesenchymal cells: bone marrow, unrestricted somatic stem cells and BFP. BFP MSCs were found to have one of the highest bone-forming abilities and, given the relatively simpler protocol for harvesting a fragment of BFP compared to other sources of MSCs, this makes the BFP an extremely valuable and easily accessible source of MSCs [39]. These data open new horizons in the development of tissue engineering and its interdisciplinary interaction with various dental disciplines [40]. 

Taking into account the possibility of BFP MSC differentiation into different types of tissues, including bone tissue, it is necessary to conduct more research for the possible development of a method of directed intraoperative differentiation of BFP cells, which opens perspectives for restoration of the lost tissue complex in the OAC zone, as well as the application of the BFP for directed bone regeneration. This will eliminate the need for additional surgical interventions, such as sinus elevator, before subsequent implant treatment.

## 5. Conclusions

During the morphological study of BFP samples, we revealed statistically significant signs of MSCs presence (*p* < 0.05), including in the brown fat tissues, which proves the high reparative potential of this type of tissue and can make the BFP a choice option among other autogenous donor materials when eliminating OAC and other surgical interventions in the maxillofacial region.

## Figures and Tables

**Table 1:** Characteristics of used antibodies.

Name of Marker	Clone	Manufacturer, Country	Dilution
CD44	SD391	Xiamen Talent Biomedical, China	RTU
CD10	GM003	PrimeBioMed, RF	1:100
CD133	-	Huabio, USA	1:500

**Table 2:** Age of patients with CD44 expression of BFP samples.

% of Expression	CD44, Amount of Samples	Min Age	Max Age	Mean Age
10	2	19	19	19.0
5	11	21	38	29.1
1	14	21	37	27.3
0	3	33	42	37.7

**Table 3:** Age of patients with CD113 expression of BFP samples.

% of Expression	CD133, Amount of Samples	Min Age	Max Age	Mean Age
10	2	19	19	19.0
5	8	25	38	29.5
1	9	21	34	26.6
0	11	21	42	30.7

**Table 4:** Age of patients with CD10 expression of BFP samples.

% of Expression	CD10, Amount of Samples	Min Age	Max Age	Mean Age
10	8	19	38	26.5
5	3	29	33	30.3
1	8	21	34	26.2
0	11	21	42	30.7

## Data Availability

The data presented in this study are available on request from the corresponding author.
